# Supplementary material for: Facultative Annual Life Cycles in Seagrasses
Source: Plants (Basel). 2023 May 16;12(10):2002. doi: 10.3390/plants12102002 (PMC10223934; doi:10.3390/plants12102002)
Supplement: Supplementary file 1 [file plants-12-02002-s001.zip › S1. updated format Supplementary Information - Seagrasses with quiescent phases 2.0.pdf]

S1. Seagrasses with vegetative quiescent phases

Environmental conditions, especially temperature, are more stable in water than on land, and changes in the aquatic environment, such as tides or floods are often predictable. A vegetative clonal growth strategy is favoured in such relatively stable environments [81], and limited sexual reproduction has been observed in many highly clonal, aquatic species which tend be perennial [82,83,84]. Especially the marine species, also named seagrasses, tend to persist through asexual clonal reproduction through extension of below-ground rhizomes, and extensive evergreen seagrass meadows can potentially be maintained largely through clonal reproduction. Thus, at the asexual end of a sexual-asexual continuum, seagrasses display meadow-forming large genets of hundreds or even thousands year old, such as those of *Posidonia australis* in Shark Bay, Australia [85], *Posidonia oceanica* in the Mediterranean [86,87,88], *Zostera marina* in the Baltic Sea [89] or *Thalassia testudinum* in the Gulf of Mexico [90] and an Estuary in Florida [91]. At the other end of this asexual-sexual reproductive continuum, populations exposed to seasonally recurring unfavourable periods of growth, depend entirely on annually recurring sexual recruitment for persistence (i.e. facultative annuals).
However, populations of some species that are seasonally or periodically exposed to unfavorable conditions for vigorous vegetative growth, pass these periods by persisting quiescent below-ground plant parts. In these cases, the seagrass populations have only visible (above-ground) tissue during part of the year, and recover through vegetative growth from persistent rhizome sections with quiescent (dormant) meristems during the favourable season for growth. In addition, these populations often depend, to a greater or lesser degree, on yearly recruitment from seeds for population maintenance. Such populations are considered perennial in this study.

Examples of vegetative quiescent seagrass species
1 *Zostera* spp.
The dwarf eelgrasses *Z. noltei*, a delicate seagrass growing in the upper intertidal shows large seasonal fluctuations in biomass and in N Europe where the plants overwinter as small rhizome fragments without leaves [92,93,94,95]. The overwintering rhizome sections or seeds initiate patches through vigorous vegetative expansion in spring, which may eventually form large continuous beds under favorable conditions. The small seeds form a seed bank for periods <1 y [92] to > 3y [95]. This is a well-studied species, and no annual populations have been found until date. The more robust eelgrass *Zostera muelleri* from eastern Australia in the lower intertidal may also have overwintering rhizomes [96,97].

2. *Halophila* spp.
Halophila australis died off completely during the cooler months in an estuary in Australia [98]; whether recovery occurred from seed or from quiescent rhizome sections was not been assessed, and requires further study. *Halophila ovalis,* coexisted with evergreen *H. beccari* and *Halodule pinnifolia*, on tidal estuarine prone to large salinity fluctuations in southern Thailand. But in contrast with the latter two species, *H. ovalis* did not resist reductions in salinity and above-ground visible plant parts were therefore absent for 6 months during the rainy season when salinities are low. When conditions become favourable (increasing salinity) during the dry season, it recruited the flats from rhizome sections and seeds [99]. Seagrasses in deeper waters are also exposed to extremes; especially reduced light. Deep water populations of *Halophila* species in Australia Great Barrier reef

presented different strategies to seasonal light reduction and disturbance; whereas *H. decipiens* had a true annual pattern, *H. spinulosa* and *H. tricostata* passed the period by quiescent rhizome sections and seeds, and *H.* ovalis had green leaves throughout the year [11]; however, these life histories of deep water *Halophila spp.* may vary with latitude [100].

3. *Ruppia* spp.

Ruppia species from the Australia and New Zealand, commonly occurring in seasonally wet shallow < 0.5m deep) hipo- or hyper saline estuarine waters or salt lakes [101]. Turions are common vegetative propagules of *Ruppia tuberosa* and *R. polycarpa* from the Australian continent and surroundings. These turions have dormant meristems and serve to survive periods of desiccation [102,103,104,105]. A third more robust ever-green Australian species *R. megacarpa* is always perennial and occurs in permanently submersed habitats, which produces less flowers and fruits than its congeneric facultative annuals [103]. The plants survive desiccation periods as long as 7-8 months year^{-1}, as either as seeds or perennating below-ground organs (turions with dormant meristems. *R. tuberosa* more so (89% of reproductive units were turions, the rest seeds) than *R. polycarpa* (86% of the reproductive units were seeds, the rest turions [103]).

4. Exceptional events:

There are various anectodical records of seagrasses that lost above-ground tissues during exceptional events, and that recovered afterwards from surviving below-ground rhizomes. Such foliar tissue loss after extreme events differs from defoliation by grazers such as sea urchins or green turtles, as the grazers usually leave the foliar meristems intact and active; although extreme grazing pressure may exploit the reserves of the plants and eventually result in seagrass loss (e.g., [106,107]). For example, *Thalassia testudinum* at Morrocoy Park Venezuela suffered from almost complete defoliation after extremely heavy rainfall lowered salinity in 1996 [108] and 1999 [109], but the seagrass canopy recovered within months from surviving rhizomes, most likely from dormant meristems [110]. *Cymodocea nodosa* populations in Israel may (almost) disappear during occasional severe winter storms, but recover by vigorous vegetative growth in spring [111,112]. *Amphibolis antarctica* suffered from widespread defoliation in Shark Bay, Australia after a marine heatwave and floods in the summers of 2010 and 2011. Recovery of foliar biomass was evident after two years, most likely from reminiscent below-ground rhizomes, although the authors do not explicitly mention this [113].
