# Supplementary material for: Facultative Annual Life Cycles in Seagrasses
Source: Plants (Basel). 2023 May 16;12(10):2002. doi: 10.3390/plants12102002 (PMC10223934; doi:10.3390/plants12102002)
Supplement: Supplementary file 1 [file plants-12-02002-s001.zip › S2 updated Supplementary Information - Facultative annual seagrass species.pdf]

Supplemental material: table facultative annual species

Table S2. Occurrence of annual and perennial growth forms of facultative annual seagrass species with characteristics of habitat and seeds. If available depth is presented for subtidal beds in parenthesis. Seeds: (P) annual production, (D) density in peak reproductive season, (B) in seed bank. *Ruppia maritima* is separated per continent, because they may be different species [114]. *Zostera japonica* is separated in East-and West Pacific because it is recently introduced in the last region. na not available, reprod shoots: reproductive (spathe-bearing) shoots.

Species	Annual/ Perennial	Location	Latitude	Environment (depth)	Most conspicuous difference with perennial population	Seeds (m^{-2})	Source	Literature reference (names)
**Hydrocharitaceae**								
Halophila beccarii								
	Annual	Malaysia, Pengkalan Nangka	6°N	Intertidal sand flat, salinity 0-25‰	Extreme heavy rainfall; reduced salinity	(P) 4814-15608*	[22]	Zakaria et al. 2002
	Perennial	Bangladesh, Bakkhali	21°N	Intertidal in saltmarsh, coexisting with *Spartina* sp.		na	[115]	Abu Hena et al. 2007
	Perennial	Vietnam, Cau Hai lagoon	16°N	Lagoon with large seasonal fluctuations in salinity- relies on seeds for maintenance		abundant	[116]	Phan et al. 2017
	Perennial	Myanmar, Kalegauk Island	15°N	Upper intertidal of mangroves swamp		na	[117]	Aye et al. 2014
	Perennial	India, Goa	14°N	Lower intertidal		na	[118]	Jagtap & Untawale, 1981
	Perennial	Thailand, Thale Sap Songkhla	7°N	Intertidal silt flat		na	[99]	Angsupanich 1996
	Perennial	Malaysia, Kemaman	4°N	Intertidal mud-flat and among roots of mangroves		(P) 156	[119]	Zakaria et al. 1999
Halophila decipiens								
	Annual	Australia, Hardy Inlet	34°S	Subtidal (2.1-2.6 m)	Reduced light (salinity)	(P) 176880	[120]	Kuo & Kirkman (1995)
	Annual	USA, Indian River Lagoon	27°N	Subtidal (2-3m), turbid		na	[121]	Kenworthy 2000
	Annual	Japan, Okinawa	26°N	Subtidal (11-23 m)		na	[122]	Kuo et al. 1995
	Annual	Florida, US	25°N	Subtidal >15 m		na	[123]	Bell et al. 2008

	Annual	Japan, Nansei Islands	24°N	Subtidal (38m)		na	[124]	Kuo et al. 2006
	Annual	Mexico, Yucatan	21°N	Subtidal (6 m)	Reduced light & salinity	na	-	Van Tussenbroek, unpubl data
	Annual	Australia, Great Barrier Reef	21°S	> 15 m depth		na	[125]	York et al. 2015
	Annual	Thailand, Trang, Khao Bae Na	7°N	Intertidal-5m depth	NA	abundant	[126]	Lewmanomont et al. 2000
	Annual	Great Barrier Reef, Green Island	16°S	Subtidal (>10-15m)	Reduced light	(B) 11000	[11]	Chartrand 2021
	Perennial	US, Florida Continental Shelf	25°N	Subtidal (10-20m)		(D) max 3628<br>(B) 134-3414	[127]	Hammerstrom et al 2006
	Perennial	Mexico, Gulf of California	24°N	Subtidal (4-6m)		(D) 41335**	[128]	Santamaría-Gallegos et al. 2006
	Perennial	Hawaii, various islands	20°N	Subtidal (1-40m)		na	[129]	McDermid et al. 2002
	Perennial	US Virgin Islands, St. Croix	18°N	Subtidal (15-27 m), clear		(D) 3530-36712	[130, 131]	Josselyn et al 1986<br>Williams 1988
	Perennial	Panama, Toro Point	9°N	Subtidal (1-1.5m), turbid		(B) 13500<br>(P) 32700***	[132]	McMillan & Soong 1989
**Fam.Ruppiaceae**								
Ruppia spiralis								
	Annual	Netherlands, Texel	53°N	One exceptional temporary wet ditch	dry in winter	na	[133]	Verhoeven 1979
	Annual	France, Camargue	44°N	Brackish ponds, falling dry in late-summer		few	[133]	Verhoeven, 1979
	Annual	NE Spain, Fra Ramon	42°N	Intertidal (0 to +0.5m)	falling dry in summer	(D) 593	[134]	Gesti et al. 2005
	Annual	S-Africa	33°S	Temporarily open estuaries, falling dry when closed		(B) 2852<br>(P) 26242	[135, 136, 137]	Riddin&Adams 2008, 2009, Vromans et al. 2013
	Perennial	Sweden, Baltic Sea	59°N	Sheltered brackish bays (0-3m), salinity 4-7 ‰		(B) 2375	[21]	Kautsky 1990
	Perennial	Denmark, Ringkøbing Fjrod	56°N	Brackish water (0.3 m)		na	[138]	Kiørboe 1980
	Perennial	Netherlands, Delta and Wadden Sea	53°N	Brackish lakes and ponds		few	[133]	Verhoeven 1979

	Perennial	France, Camargue	44°N	River delta		few	[133]	Verhoeven, 1979
	Perennial	NE-Spain, Fra Ramon	42°N	Permanently flooded (0-0.3m)		0-several	[134]	Gesti et al. 2005
	Perennial	Spain, Ebro Delta	41°N	Permanently flooded		na	[139, 140]	Menéndez & Comín 1989, Menéndez 2002
	Perennial	SE-Spain, Murcia	38°N	Shallow		na	[141]	Ballester 1985
	Perennial	Portugal, Santo Andre Lagoon	38°N	Shallow (1.5m)		na	[142]	Calado & Duarte 2000
	Perennial	Tunesia, Bizerte Lagoon	37°N	Shallow		na	[143]	Casagranda & Boudouresque 2007
Ruppia maritima								
**Europe/Africa**	Annual	Baltic, Sweden	59°N	Brackish waters (0-3m), winter die off, salinity 4-7‰		na	[21]	Kautsky 1990
	Annual	Netherlands, Delta and Wadden Sea	53°N	Brackish lakes and ponds		abundant	[133]	Verhoeven 1979
	Annual	France, Camargue	44°N	Brackish pools	falling dry in summer	abundant	[133]	Verhoeven, 1979
	Annual	Mediterranean, Camargue	43°N	Marshes, temporarily falling dry		(B) 2920-5030	[144]	Bonis et al. 1995
	Annual	Greece, Evros Delta	41°N	Temporal flooded lagoon	temporarily falling dry	na	[145]	Malea et al 2004
	Perennial	Netherlands, Delta & Wadden	53°N	Brackish lakes and ponds		abundant	[133]	Verhoeven 1979
	Perennial	France, Camargue	44°N	Permanent pool		abundant	[133]	Verhoeven, 1979
	Perennial	Greece, Evros Delta	41°N	Permanent lagoon		na	[145]	Malea et al. 2004
**American continent**	Annual	Canada, British Columbia	49°N	Intertidal river delta		(P) 1680-4910	[146]	Bigley 1981
	Annual	USA, New Hampshire	43°N	Shallow pools	falling dry in summer	na	[147]	Richardson, 1980
	Annual	Argentina, Rio Colorado	39°S	River mouth, irrigation network	dried out for maintenance during winter	(B) 4352	[148]	Acosta et al. 1999
	Annual	USA, Chesapeake Bay	37°N	Salt pond	desiccation in late summer (0-0.3 m)	(D) 2188-15480	[149]	Silberhorn 1996

	Annual	Brazil, Patos Lagoon System	32°S	Shallow sub-tidal in open Estuary. Dry period Nov-Jan		na	[150]	Koch & Seeliger 1988
	Annual	USA, Gulf of Mexico, Alabama	30°N	Estuary (0.3-0.6 m), late-summer senescence and uprooting		(B) 1193-31809	[151]	McGovern 2009
	Annual	USA, Guadeloupe Estuary, Texas	28°N	Estuary (0.3m), 4 months-growth cycle, higher nutrients, lower salinity	Reduced light or wave-exposure in winter	abundant	[152]	Dunton 1990
	Annual	Mexico, Pacific Verde Lagoon	23°N	Inlet, highly fluctuating salinity, erosion in rainy season due to high river flow		na	[153]	Flores-Verdugo et al. 1988
	Perennial	USA, New Hampshire	43°N	Deeper permanently flooded habitats		na	[147]	Richardson, 1980
	Perennial	Argentina, Rio Colorado	39°S	River mouth, permanently submersed drainage canals		(B) 200	[148]	Acosta et al. 1999
	Perennial	USA, Chesapeake Bay	37°N	Estuary, river-mouth (1.2-1.4 m)		(D) 2430-29670	[149, 154]	Silberhorn 1996, Rosenzweig & Parker 2002
	Perennial	Brazil, Patos Lagoon System	32°S	Shallow sub-tidal (0.2-0.7 m)		na	[150, 155]	Koch & Seeliger 1988, Texeira da Silva & Asmus 2001
	Perennial	USA, Louisiana, Lake Pontchartrain	30°N	Shallow estuary, low salinity		(D) 500 - 20000	[156]	Cho & Poirrier 2005
	Perennial	USA, Nueces Estuary, Texas	28°N	Bay (0.1-0.5m)		(D) 4110	[157, 158]	Pulich 1985, McMillan 1985
	Perennial	USA, Tampa Bay	28°N	Estuary, tidal flat & shallow subtidal		na	[159]	Lazar & Dawes 1991
	Perennial	USA, Everglades /Florida Bay	25°N	Estuary (< 1m)		(P) 150-1783	[160]	Strazisar et al. 2013
	Perennial	Mexico, Pacific, Bahia Magdalena	22-27°N	Shallow lagoon		na	[161]	Lopez-Calderon et al. 2010
	Perennial	Mexico, Caribbean, Nichupté	21°N	Brackish-saline lagoon		abundant		Van Tussenbroek, unpubl data
**Australia**	Perennial	Australia, Blackwood River	34°S	Estuary		0	[162]	Congdon & McComb 1979
**Zosteraceae**								

Zostera japonica								
West Pacific	Mixed annual	China, Swan Lake	37°N	Intertidal	Ice cover and lower winter temperatures	40244	[163	Zhang et al. 2020
	Perennial	China, Swan Lake	37°N	Intertidal		22228	[54]	Yue et al. 2020
	Perennial	China, Swan Lake	37°N	Intertidal		21630	[54]	Yue et al. 2020
	Perennial	W-Korea	37°N	Intertidal		na	[164]	Lee et al. 2005
	Perennial	China, Huiquan Bay	36°N	Intertidal		12501	[163]	Zhang et al. 2020
	Perennial	China, Huiquan Bay	36°N	Intertidal		7459	[54]	Yue et al. 2020
	Perennial	China, Huiquan Bay	36°N	Intertidal		2821	[54]	Yue et al. 2020
	Perennial	S-Korea	35°N	Intertidal		na	[165]	Lee et al. 2006
	Perennial	Japan, Mikawa Bay	35°N	intertidal		na	[166]	Nakaoka & Aioi 2001
	Perennial	S-Korea	35°N	intertidal		0	[167]	Park et al. 2011
	Perennial	S-Korea	35°N	intertidal		7850	[59]	Suonan et al. 2017
	Perennial	S-Korea	35°N	intertidal		6220	[59]	Suonan et al. 2017
	Perennial	S-Korea	35°N	intertidal		1560	[59]	Suonan et al. 2017
	Perennial	Hong Kong	22°N	Intertidal		na	[168]	Lee 1997
	Perennial	N-Vietnam	21°N	intertidal		na	[169]	Huong et al. 2003
East Pacific	Annual	Canada, British Columbia	49N	Intertidal		70% of shoots flowering	[20]	Harrison & Bigley 1982
	Annual	Canada, British Columbia	49°N	Intertidal		34060	[146]	Bigley 1981
	Perennial	USA, Washington	47°N	Intertidal (0.1 - 1.5 m above MLLW)		na	[170]	Ruesink et al. 2010
	Perennial	USA, Oregon	45°N	Intertidal		(D) 49-461 reprod shoots	[171]	Kaldy 2006
Zostera marina								
	Annual	Bahia Concepción Gulf of California	26 °N	Subtidal (2-4 m)	Heat stress	30000	[42]	Santamaria-Gallegos et al. 2000
	Annual	Bahia Concepción Gulf of California	26 °N	Subtidal		(P) 17000	[172]	Lopez-Calderon et al. 2016
	Annual	Infiernillo Channel Gulf of California	28 °N	Subtidal		(P) 6211 – 71187 (or 605 71605 mean 17442)	[172]	Lopez-Calderon et al. 2016
	Annual	Mexico, Gulf of California	29°N	Subtidal (2 to 5m)	Heat stress	(P) 41998-100376	[14]	Meling-Lopez & Ibarra-Obando 1999

	Annual	Gulf of California	29 °N	Subtidal (depth unknown)		19783	[28]	Phillips et al. 1983
	(Mixed) Annual	Chesapeake Bay, North Carolina USA	34 °N	Subtidal, 0.20-0.50m	Temperature, salinity, DO, chla, more muddy and organic	(Ppotential) 61563<br>(Pmax) 12699<br>(B) 190-906	[13]	Jarvis et al. 2012
	Annual	Ago Bay, Japan	34 °N	Subtidal (1-4m) mud	Muddier	(B) 219-1157<br>(P) 6000	[173]	Morita et al. 2007
	Annual	Hamana-Ko, Japan	34°N	Subtidal (3 – 3.5 m)	Low salinity and higher maximum temperature in inner part of lagoon	na	[174 in 166]	Imao &Fushimi 1985 in Nakaoka & Aioi 2001
	Annual	S-Korea, Jindong Bay	35°N	Subtidal (4-7m)	Depth/light limitation	(B) 2138-3120<br>(P) 8223-9981	[40]	Kim et al. 2014
	Annual	Moon Lake, China	37 °N	Intertidal	Disturbance by clam harvesting and exposure. Desiccation also limits (P)	(P) 416<br>(B) 112	[43]	Qin et al. 2014. This population does not seem to be truly annual; seeds may originate of the perennial site.
	Annual	Rhode Island, USA	41 °N	Subtidal, 0.30 m	Water dynamics and burial	na	[37]	Harlin et al. 1982
	Annual	Yaquina, Oregon, USA	44 °N	Intertidal, +0.50 - +1.50 (above MLT)	Temperature , desiccation (lie flat)	(D repr shoots) 164	[29, 175]	Bayer 1979, Boese et al. 2005
	Annual	Nova Scotia, Canada	44 °N	Intertidal (0.5 to 2 m above MLT)	Desiccation	(P) 78224	[7, 176, 30]	Keddy & Patriquin 1978, Robertson & Mann 1984, Keddy 1987
	Annual	Brittany France	48 °N	Intertidal	Extreme fluctuations, desiccation	na	[44]	Becheler et al. 2010. Note, this population is ephemeral (not present every year) MvK pers obs. Probably from seed of the nearby population
	Annual	Lake Veere, The Netherlands	51 °N	Submersed (0.70 m)	Macroalgae, grazing, storms,	(P) 46261-60074<br>(B) 664-864	[36, 39]	Van Lent & Verschuure 1994a, b

					light limitation, low salinity			
	Annual	Oosterschelde, The Netherlands	51 °N	Intertidal (0.90 m above MLT)	Macroalgae, grazing, storms, desiccation	(P) 26764-50214<br>(B) 128-376	[36, 35]	Van Lent & Verschuure 1994a, Harrison 1993
	Annual	Oosterschelde, The Netherlands	51 °N	Intertidal (0.90 m above MLT)		(D) 200	[92]	Hootsmans et al. 1987
	Annual	Wadden Sea, The Netherlands	53 °N	Intertidal (0.80 m above MLT)	Algal cover, desiccation	(B) 5-40	[177]	van Katwijk et al. 2010
	Annual	De Bol, The Netherlands	53 °N	Submersed (0.75 m)	(low salinity), algal cover	na	[38]	Verhoeven & van Viersssen 1978
	Annual	Wadden Sea, Ems Est, The Netherlands	53 °N	Intertidal		(P) 300	[178]	Erftemeijer et al. 2008
	Annual	Wadden Sea, Germany	55 °N	Intertidal (0.80 m above MLT)	Macroalgae, grazing, storms, ice	(B) 60	[177]	van Katwijk et al. 2010
	Perennial	Mexico, Baja California	26°N	Intertidal and shallow subtidal		(D) 50-600 reprod shoots	[179]	Cabello-Pasini et al. 2003
	Perennial	Mexico, Baja California	30°N	Intertidal and shallow subtidal		(D) 1561-3969	[180]	Poumian-Tapia & Ibarra-Obando 1999
	Perennial	Chesapeake Bay, North Carolina USA	34 °N	Subtidal, 0.20-0.50m		(Ppotential) 41146<br>(Pmax) 8515<br>(B) 147-211	[13]	Jarvis et al. 2012
	Perennial	Ago Bay, Japan	34 °N	Subtidal (3-12m), sand		(B) 5-21	[173]	Morita et al. 2007
	Perennial	Hamana-Ko, Japan	34 °N	Subtidal (1.5 m)		na	[174 in 166]	Imao &Fushimi 1985 in Nakaoka & Aioi 2001
	Perennial	S-Korea, Jindong Bay	35°N	Subtidal (2m)		(B) 1834-3274<br>(P) 6024-7114	[40]	Kim et al. 2014
	Perennial	S-Korea	35 °N	Subtidal (5m)		(P) 25476	[181]	Lee et al. 2007
	Perennial	Huiquan Bay	36 °N	Subtidal		(P) 29000 +- 10658<br>(B) 35 - 330	[182]	Xu et al. 2018
	Perennial And annual	Swan Lake, China (= Moon Lake)	37 °N	Subtidal		(P) 53624 +- 19628<br>(B) 0-552	[182]	Xu et al. 2018

	Perennial	Moon Lake, China	37 °N	Intertidal and subtidal		(P) 3820-60793<br>(B) 77-584	[43]	Qin et al. 2014
	Perennial	Chesapeake Bay, North Carolina USA	37 °N	Subtidal 0.5-0.7m		(B) 0-91	[183]	Jarvis & Moore 2010
	Perennial	Chesapeake Bay, North Carolina USA	37 °N	Subtidal 0.2-0.4m		(B) 0-41	[183]	Jarvis & Moore 2010
	Perennial	Chesapeake Bay, North Carolina USA	37 °N	Subtidal 0.1-0.8m		(B) 0-33	[183]	Jarvis & Moore 2010
	Perennial	Chesapeake Bay, US	37 °N	Subtidal (0.5-1.5m)		(P) 8127	[184]	Silberhorn et al. 1983
	Perennial	Chesapeake Bay, US	Ca. 37 °N	Subtidal >0.3m		(B) 617<br>(Repr)839	[185]	Harwell & Orth 2002
	Perennial	Bohai Sea, China	40 °N	Subtidal		(P) 1020	[52]	Xu et al. 2020
	Perennial	Bohai Sea, China	40 °N	Subtidal		(P) 830	[52]	Xu et al. 2020
	Perennial	Yaquina, Oregon, USA	44 °N	Subtidal to Intertidal (0.50 m above MLT)		na	[29, 175]	Bayer 1979, Boese et al. 2005
	Perennial	Nova Scotia, Canada	44 °N	Low intertidal and subtidal (0.5 above LT -1m)		(P) 11328	[7, 176, 30]	(Keddy & Patriquin 1978, Robertson & Mann 1984, Keddy 1987)
	Perennial	Brittany France	47°N 48 °N	Low-intertidal and subtidal		na	[44]	Becheler et al. 2010
	Perennial	Lake Grevelingen, The Netherlands	51 °N	Submersed (0.60 m)		(P) 641-13463<br>(B) 56-104	[36]	Van Lent & Verschuure 1994a
	Perennial	Lake Grevelingen, The Netherlands	51 °N	Submersed (1.25 m)		(P) 500-14876<br>(B) 184-320	[36]	Van Lent & Verschuure 1994a
	Perennial	Odense Fjord, Baltic Sea	55 °N	Submersed (1-3 m)		(B) 11200 (7543-16951) of which 978 in upper 6 cm	[186]	Greve et al. 2005
	Perennial	South Funen, Denmark	55 °N	Submersed 6 m		(P) 753	[187]	Olesen et al. 2017
	Perennial	South Funen, Denmark	55 °N	Submersed 4 m		(P)1233	[187]	Olesen et al. 2017
	Perennial	South Funen, Denmark	55 °N	Submersed 1.8 m		(P) 1973	[187]	Olesen et al. 2017
	Perennial	Limfjorden, Denmark	56 °N	Submersed (no tides) 0.7m		(P) 3393-17550	[188]	Olesen 1999

*Variation between years: from fruit production assuming 1.7 seeds/fruit [119]

**From fruit density: assuming 35 seeds per fruit [130]

***Sum of monthly seed bank
