# Supplementary material for: Facultative Annual Life Cycles in Seagrasses
Source: Plants (Basel). 2023 May 16;12(10):2002. doi: 10.3390/plants12102002 (PMC10223934; doi:10.3390/plants12102002)
Supplement: Supplementary file 1 [file plants-12-02002-s001.zip › S3 updated format Supplementary Information Zostera marina.pdf]

Supplementary Information 3. Table with reproductive traits of *Zostera marina* populations, comparing annual populations with the nearest perennial population. For the first two populations no nearby perennial population exists (distance > 1000km). *C*oloring refers to types of environment conform Figure 1: Pink: Environments with yearly recurrent heat stress and high salinity. Purple: Subtidal or permanently submersed environments experiencing anoxia-related stress. Blue: Mid-intertidal environments with twice-daily exposure to air.

no	Location	Lati-tude	Emersion	Distance nearby perennial pop. km	Habitat difference annual as compared to nearby perennial population	%reproductive shoots (at end of growing season) except **	variable	annual population	nearest perennial population	factor annual / perennial	source
1	Bahia Concepcion, Gulf of California	26 °N	subtidal	1000		100%	potential seed production m^{-2}	43300			[42, 64]
2	Infernillo Channel, Gulf of California	29 °N	subtidal	1300		100%	max seed production m^{-2}	78224			[14,28]
3	Chesapeake Bay, North Carolina USA	34 °N	subtidal	15	Temp >, salin <, DO <, chla >, muddier & more % org. matter	33%	max seed production m^{-2}<br>max seed density seedbank m^{-2}	61563<br>906	41146<br>33	1.50<br>27.45	[13]
4	Ago Bay, Japan	34 °N	subtidal	circa 6	shallower, muddier, summer temp >, winter temp <, salin <	43-59%	max seed density seedbank m^{-2}	1157	21	55.10	[173]
5	Hamana-Ko, Japan	34 °N	subtidal	circa 5	Low salinity and higher max temperature in inner part of lagoon	Ann 100%<br>Per 20%	NA				[166]
6	Jindong Bay, South Corea	35 °N	subtidal	0	deeper, darker	92-96	max seed production m^{-2}<br>max seed density seedbank m^{-2}	9981<br>3120	7114<br>3274	1.40<br>0.95	[40]
7	Rhode Island, USA	41 °N	subtidal	0	more sand, lower organic content, shallower	100	NA				[37,189]
8[b]	Lake Veere, Netherlands[a]	51 °N	Perm. submersed	30	more macroalgae, darker, lower salinity	Ann 53-59%**<br>Per 4-47%**	max seed production m^{-2}<br>max seed density seedbank m^{-2}	60074<br>864	14876<br>320	4.04<br>2.70	[36,39, 34]
9[b]	Bol, The Netherlands [a]	53 °N	Perm. submersed	175	lower salinity, probably more macroalgae		NA				[38]
10	Moon Lake, China	37 °N	Intertidal[4]	0	emersion*, more clam harvesting		max seed production m^{-2}<br>max seed density seedbank m^{-2}	416<br>112	60793<br>584	0.01<br>0.19	[43]
11	Yaquina Bay, USA	44 °N	Intertidal[4]	0	emersion*	100					[29,175]
12	Nova Scotia, Can.	44 °N	intertidal[4]	0	emersion*	100	max seed production m^{-2}	78224	11328	6.91	[30,7, 176]
13	Oosterschelde, Netherlands [a]	51 °N	intertidal	30	emersion*	Ann 40-49%**<br>Per 4-47%**	max seed production m^{-2}<br>max seed density seedbank m^{-2}	50214<br>376	14876<br>320	3.38<br>1.18	[36,39, 35,92]
14[b]	Wadden Sea Balgzand, NL	53 °N	intertidal[4]	330 (1.5)[1]	emersion*	26%	NA				[190,177]
15[b]	Wadden Sea Terschelling, NL	53 °N	intertidal[4]	370 (10)[2]	emersion*	50-75%	NA				[177,34, 31]
16	Wadden Sea Ems, Netherlands	53 °N	intertidal[4]		emersion*	77-100%					[178]
17	Wadden Sea, Sylt, Germany	55 °N	intertidal[4]	250 (1.5)[3]	emersion*	45-65%					[177]
AVERAGE FACTOR ANNUAL/PERENNIAL										8.73	

(continued)

[a] Nearest perennial is assumed to be Lake Grevelingen, Netherlands (permanently submersed, no tides); seagrass disappeared during 1990s.

[b] Population no. 8, 9, 14 & 15 as well as the reference perennial Grevelingen have disappeared between the 1970s and 2000s

[1]presently probably Thames Estuary, 330 km; before the wasting disease 1930s: 1.5 km

[2]presently probably Thames Estuary 370 km; before the wasting disease 1930s: 10 km

[3]presently probably Baltic 250 km through canal; before the wasting disease 1930s: 1.5 km

[4]perennials (used to) grow low intertidal and subtidal

*emersion during low tide may lead to desiccation, more waterfowl grazing, more temperature fluctuations (e.g. Bayer 1979)

**biomass percentage, not shoot percentage
